# Evaluating the Impact and Practicality of a National Digital Intervention for Type 2 Diabetes Mellitus: Single-Arm Nonrandomized Pilot Trial

**DOI:** 10.2196/94551

**Published:** 2026-07-29

**Authors:** Alice Moi Ling Yong, Hiu Nam Chan, Pui Lin Chong, Chee Kwang Yung, Musjarena Mulok, Syuhrah Musa, Si Yee Chan, Yvonne Lee, Athirah Yusof, Yufan Chen, Hong Shen Lim

**Affiliations:** 1 Endocrine Centre, Raja Isteri Pengiran Anak Saleha (RIPAS) Hospital, Jalan Putera Al-Muhtadee Billah, Bandar Seri Begawan, BA1712, Brunei Darussalam, +673 2242424; 2 EVYD Research Pte Ltd, Singapore, Singapore; 3 Department of Health Services, Ministry of Health, Bandar Seri Begawan, Brunei Darussalam; 4 EVYD Technology Sdn Bhd, Bandar Seri Begawan, Brunei Darussalam

**Keywords:** type 2 diabetes mellitus, telemedicine, intervention, lifestyle modification, quality of life

## Abstract

**Background:**

Type 2 diabetes mellitus (T2DM) requires sustained self-management and lifestyle modification to achieve optimal glycemic control. Hybrid care models that combine digital health technologies with in-person clinical support may enhance patient engagement while improving the accessibility and scalability of diabetes care. However, evidence regarding their effectiveness and feasibility in Southeast Asian populations remains limited.

**Objective:**

This study aimed to evaluate the preliminary effectiveness and feasibility of a national hybrid digital intervention for individuals with T2DM in Brunei Darussalam. The primary objective was to evaluate the proportion of participants achieving a reduction in glycated hemoglobin (HbA_1c_) of ≥0.6% after 16 weeks. Secondary objectives included evaluating changes in metabolic parameters, anthropometric outcomes, and health-related quality of life (QoL).

**Methods:**

This single-arm, nonrandomized pilot trial enrolled adults with T2DM into a 16-week hybrid digital intervention integrating remote health coaching, structured digital education, self-monitoring activities, and asynchronous communication via WhatsApp. Participants attended scheduled video consultations (VCs) and submitted self-monitoring records throughout the intervention period. Clinical outcomes included changes in HbA_1c_, fasting blood glucose, lipid profile parameters, BMI, waist circumference, and QoL measured using the EQ-5D-5L instrument. Feasibility outcomes included intervention completion, VC attendance, participant engagement, and intervention acceptability.

**Results:**

A total of 122 participants were enrolled, and 108 (88.5%) completed the intervention. Among 104 participants with complete HbA_1c_ data, there was a mean reduction of 1.2% (95% CI −1.45 to −0.96; *P*<.001). Overall, 65.4% (68/104) of participants achieved the predefined HbA_1c_ reduction threshold of ≥0.6%, while 84.6% (88/104) demonstrated an overall reduction in HbA_1c_. Significant improvements were also observed in fasting blood glucose (−1.7 mmol/L, 95% CI −2.3 to −1.2; *P*<.001), BMI (−0.4 kg/m², 95% CI −0.6 to −0.2; *P*<.001), waist circumference (−1.9 cm, 95% CI −2.9 to −0.9; *P*<.001), total cholesterol (−0.3 mmol/L, 95% CI −0.6 to −0.2; *P*<.001), triglycerides (−0.5 mmol/L, 95% CI −0.7 to −0.2; *P*<.001), and EuroQol Visual Analog Scale (EQ-VAS) scores (+6.7 points, 95% CI 3.9 to 9.6; *P*<.001). The intervention demonstrated favorable feasibility outcomes, including an 88.5% (108/122) completion rate, attendance at ≥5 VCs by 75.9% (82/108) of participants, and high participant satisfaction, with 86.9% (86/99) of respondents reporting satisfaction or extreme satisfaction with the intervention.

**Conclusions:**

The pilot trial demonstrated the acceptability, preliminary effectiveness, and feasibility of a national hybrid digital intervention for individuals with T2DM in Brunei Darussalam. High completion rates, sustained participant engagement, and positive participant feedback support the practicality of implementation within a national digital health ecosystem. The intervention was also associated with improvements in glycemic control, metabolic outcomes, anthropometric measures, and health-related QoL. Larger controlled studies are warranted to evaluate long-term effectiveness, scalability, and implementation outcomes.

## Introduction

### Background

Type 2 diabetes mellitus (T2DM) remains one of the leading global public health challenges and is associated with substantial morbidity, premature mortality, and reduced quality of life (QoL) [1,2]. Effective diabetes management requires sustained lifestyle management, including dietary modification, regular physical activity, weight management, and self-monitoring behaviors to improve glycemic outcomes and reduce diabetes-related complications [3,4]. However, consistent long-term delivery of this routine clinical practice into interventions remains difficult due to limited health care resources, workforce constraints, and dependence on repeated face-to-face counseling [5].

The rapid expansion of digital health technologies, particularly with the COVID-19 pandemic, has accelerated the adoption of digital therapeutics (DTx) and mobile health (mHealth) interventions for chronic disease management. DTx are evidence-based therapeutic interventions driven by software programs to prevent, manage, or treat medical disorders [6,7]. In T2DM management, digital interventions commonly incorporate the use of smartphone apps, wearable devices for monitoring, telehealth consultations, remote monitoring, and personalized health coaching to support self-management and improve active patient engagement [8,9].

Digital health interventions have increasingly demonstrated potential to improve glycemic outcomes and diabetes self-management among individuals with T2DM. Systematic reviews evaluating mHealth applications, telemonitoring, and technology-enabled diabetes self-management interventions have reported improvements in glycated hemoglobin (HbA_1c_), self-management behaviors, and patient engagement [8-10]. Hybrid care approaches integrating remote support with lifestyle education have additionally shown potential to improve accessibility and continuity of chronic disease management [11,12].

Despite increasing evidence in support of DTx for T2DM management, implementation in Southeast Asia and Muslim-majority countries remains limited. Variations in digital literacy, cultural practices, health care infrastructure, and patient engagement behaviors influence the feasibility and effectiveness of digital interventions across populations. Consequently, real-world implementation studies are needed to evaluate the applicability and preliminary effectiveness of DTx within localized health care systems.

Brunei Darussalam is a small Southeast Asian country with a population of approximately 450,000 and universal access to government-funded health care services [13]. Despite this, diabetes and related metabolic risk factors remain a significant public health concern. Recent national surveillance data estimated that 15% of adults are living with diabetes, highlighting the substantial disease burden within the local population. In addition, diabetes is one of the leading causes of death, placing considerable strain on long-term health care utilization and economic resources [14].

Brunei’s national mobile app, BruHealth, has evolved from a COVID-19 contact tracing tool into a nationwide digital population health platform capable of supporting access to personal health records and health navigation features such as appointment booking. This digital infrastructure provides an opportunity to support scalable and accessible DTx delivery for T2DM management within the Bruneian health care system while potentially reducing reliance on in-person consultations and optimizing health system use.

Evidence evaluating the feasibility and effectiveness of integrated digital diabetes interventions in Brunei remains limited. The DEsireD (Development and Exploration of Effectiveness and Feasibility of Digital Intervention for Type 2 Diabetes Mellitus) study was designed to evaluate a hybrid digital intervention integrating remote health coaching, structured educational support, and self-monitoring activities for individuals with T2DM in Brunei Darussalam [15].

### Aims of This Study

This study aimed to evaluate the preliminary effectiveness and feasibility of a hybrid digital intervention for individuals with T2DM in Brunei Darussalam. The intervention combined digital lifestyle management delivered online with offline health coaching and monitoring support.

The primary objective was to evaluate the proportion of participants achieving a reduction in HbA_1c_ of ≥0.6% after 16 weeks of sustained lifestyle modifications administered through a digital intervention. Secondary objectives included evaluating changes in metabolic parameters, including fasting blood glucose (FBG) and lipid profile parameters (total cholesterol, low-density lipoprotein cholesterol [LDL-C], high-density lipoprotein cholesterol [HDL-C], and triglycerides); anthropometric parameters, including BMI and waist circumference; and health-related QoL measured using the EQ-5D-5L instrument.

## Methods

### Study Design

This study was a single-arm, nonrandomized pilot trial evaluating the feasibility and effectiveness of a hybrid digital intervention for individuals with T2DM in Brunei Darussalam. The intervention combined remote health coaching, structured digital education, and self-monitoring activities delivered through WhatsApp-supported communication. Participants were instructed to continue their existing medications throughout the study period, and no changes to prescribed treatments were made as part of the intervention; however, clinically indicated adjustments were permitted in cases of recurrent hypoglycemia for patient safety. The detailed study methodology has been published previously [15].

### Recruitment

Participants were recruited through both online and offline outreach methods. Online recruitment included in-app banners and push notifications within the BruHealth app, and social media advertisements. Offline recruitment involved flyer distribution, banners, posters displayed at health centers, and radio broadcasts.

Interested individuals were invited to contact the study team for further information and eligibility screening. Written informed consent was obtained from all participants before participation in the study.

### Eligibility Criteria

Eligibility criteria were established to identify adults with suboptimally controlled T2DM who were suitable for participation in a lifestyle-based digital intervention study. Exclusion criteria were established to minimize safety risks and ensure suitability for participation in a remotely delivered lifestyle intervention.

Textbox 1 summarizes the study inclusion and exclusion criteria.

Textbox 1. **Study inclusion and exclusion criteria.**

**Inclusion criteria**

- Diagnosed with type 2 diabetes mellitus
- HbA_1c_ (glycated hemoglobin) ≥7% within the previous 12 months
- Aged 20-70 years
- BMI 23-50 kg/m²

**Exclusion criteria**

- Pregnancy or breastfeeding
- Insulin or injectable noninsulin therapy use
- History of hypoglycemic or hyperglycemic crisis within the previous 6 months
- Blood pressure ≥160/100 mm Hg
- Recurrent acute pancreatitis
- Decompensated liver cirrhosis
- Estimated glomerular filtration rate <60 mL/min/1.73 m²
- Recent cardiovascular or cerebrovascular events within the previous 12 months
- Arrhythmias or New York Heart Association class II-IV heart failure
- Proliferative diabetic retinopathy
- Foot ulcer or gangrene
- Deep vein thrombosis or intermittent claudication
- Active cancer
- Posttransplant status or planned surgery within 6 months
- Thyroid disorders including subclinical disease
- Musculoskeletal conditions limiting physical activity
- Inability to perform activities of daily living
- Inability to use mobile social media apps (eg, WhatsApp and YouTube)

### Intervention Delivery

Participants enrolled in a 16-week hybrid digital intervention study that provided both structured digital education and offline diabetes management support provided by health care personnel. These health care personnel underwent modular training specific to the study protocol, including diabetes self-management education, communication workflows, participant engagement procedures, and escalation processes. The clinical study team consisted of health coaches, dietitians, general practitioners, and endocrinologists who collaboratively supported monitoring and follow-up throughout the intervention period. Detailed intervention workflows, escalation procedures, and multidisciplinary operational structures and roles have been described previously in the published DEsireD protocol paper [15].

Communication between participants and health coaches was conducted primarily through WhatsApp via text messages, video consultations (VCs), reminders, and submission of self-monitoring records. Participants attended a total of 7 scheduled VCs throughout the 16-week intervention period.

Weekly digital educational materials were provided through multimedia materials shared via WhatsApp and Google Drive links. Educational content focused on diabetes self-management, exercise planning, healthy dietary methods, blood glucose monitoring, and SMART (Specific, Measurable, Achievable, Relevant, and Time-bound) goal setting.

Participants self-reported dietary intake, physical activity, body weight, and waist circumference logs using paper-based diary cards. Completed records were submitted weekly by photographing and sharing the diary cards with health coaches through WhatsApp. Dietary and physical activity recommendations were provided during consultations, through participant-initiated WhatsApp inquiries, or through self-directed learning from educational materials. Participant progress, adherence, and records were reviewed through a biweekly reporting workflow between all operational personnel to support monitoring, participant follow-up, and escalation where required.

To support participant engagement with the study and facilitate sustained participation throughout the intervention, weekly reminders for diary card submission were sent every Monday through WhatsApp. Additional reminders for scheduled consultations were sent 3 days and 1 day before each consultation. To further ensure seamless digital communication, participants were provided mobile data support throughout the study period.

### Outcome Measures

The primary outcome was the proportion of participants achieving a HbA_1c_ reduction of ≥0.6% following the 16-week intervention. A reduction threshold of ≥0.6% was selected based on prior mHealth and digital intervention literature and studies reporting weighted mean HbA_1c_ reductions ranging from −0.4% to −0.9% [10,11,16].

Secondary outcomes were to evaluate changes in metabolic parameters, including FBG and lipid profile parameters (total cholesterol, LDL-C, HDL-C, and triglycerides); changes in anthropometric parameters, including BMI and waist circumference; and health-related QoL measured using the EQ-5D-5L instrument.

Feasibility and engagement outcomes included intervention completion, attendance at scheduled VCs, submission of self-monitoring records throughout the intervention period, and participant satisfaction with the intervention.

### Statistical Analysis

Statistical analyses were performed using GraphPad Prism software (GraphPad Software Inc). Continuous variables were presented as mean (SD), while categorical variables were presented as frequencies and percentages. Analyses for each outcome were performed using available participant data at both baseline and end point. Changes between baseline and end point outcomes were evaluated using paired 2-tailed *t* tests for continuous variables. Changes in paired categorical EQ-5D-5L domain outcomes between baseline and end point assessments were evaluated using McNemar tests.

As this study was conducted as a clinical trial involving multiple outcome measures, a more stringent threshold for statistical significance (*P*<.001) was applied to enhance the robustness of the findings and reduce the likelihood of type I error arising from multiple comparisons. Based on this prespecified significance threshold, findings with *P* values ≥.001 and <.05 were interpreted as not meeting the predefined threshold for statistical significance.

### Ethical Considerations

The study was conducted in accordance with the principles of the Declaration of Helsinki and Good Clinical Practice guidelines as defined by the International Council for Harmonization. Ethical approval was obtained from the Medical and Health Research and Ethics Committee, Ministry of Health, Brunei Darussalam (reference number: MHREC/MOH/2022/4[1]).

All participants received a participant information sheet detailing the study procedures, interventions, and potential risks prior to enrollment. Written informed consent was obtained from all participants before participation in the study. Participants were informed that their participation was voluntary and that they could withdraw from the study at any time without affecting their standard medical care.

## Results

### Participant Flow and Baseline Characteristics

**Figure 1.**
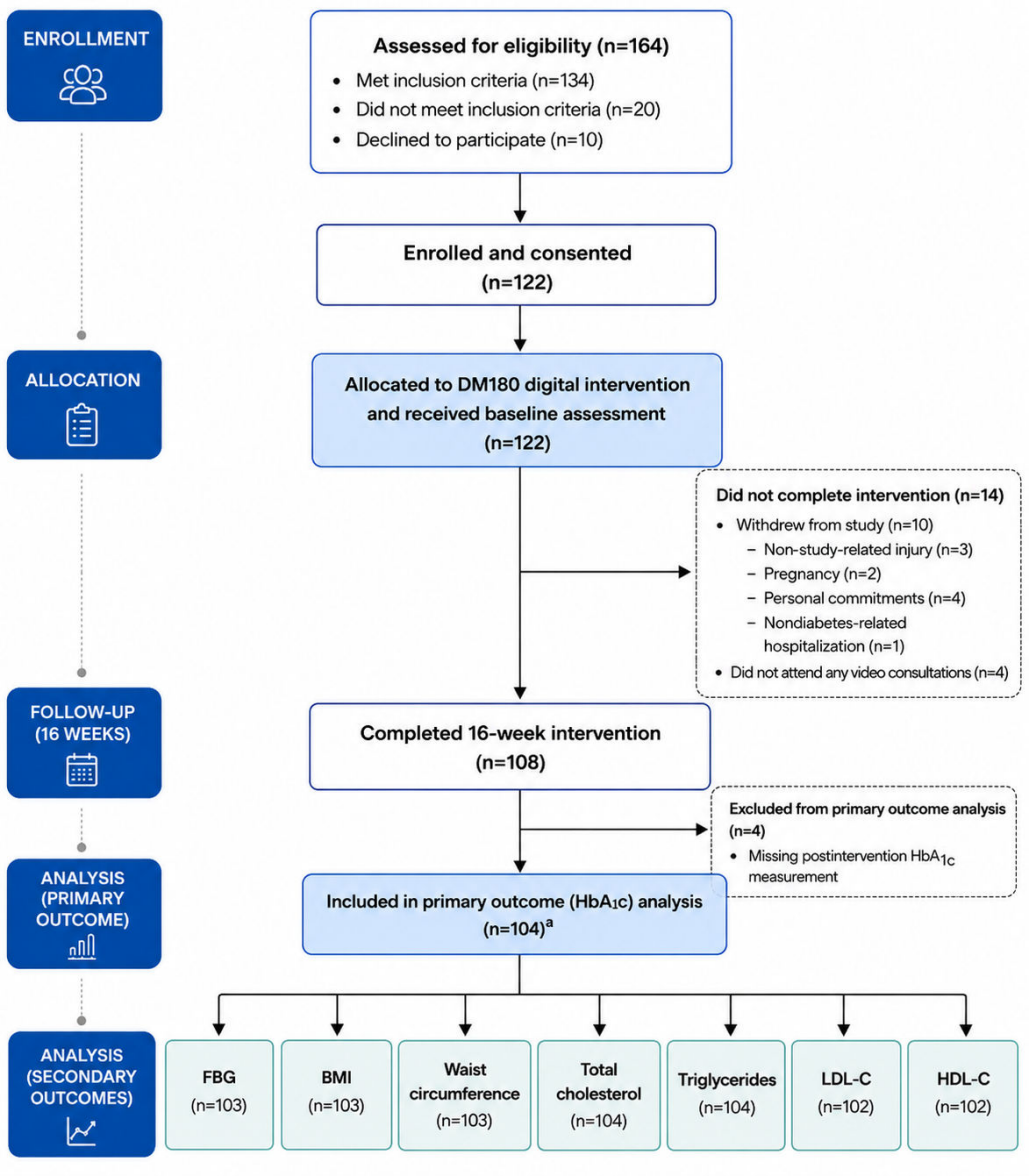
CONSORT (Consolidated Standards of Reporting Trials)-style participant flow diagram. ^a^LDL-C and HDL-C values were unavailable for 2 participants because of elevated triglyceride levels precluding calculation. One participant did not complete end point FBG assessment, and 1 participant did not submit end point anthropometric measurements. DM180: Diabetes management 180; FBG: fasting blood glucose; LDL-C: low-density lipoprotein cholesterol; HbA_1c_: glycated hemoglobin; HDL-C: high-density lipoprotein cholesterol.

A total of 122 participants were enrolled in the study. During the 16-week intervention period, 10 (8.2%) participants withdrew from the study. Four (3.3%) participants did not attend any health coach VCs but were still included in baseline and end point analyses. Overall, 108 (88.5%) participants completed the intervention. The participant flow from eligibility assessment to outcome analysis is shown in Figure 1.

Primary HbA_1c_ outcomes were available for 104 participants. Four participants who completed the intervention did not complete the postintervention HbA_1c_ assessment and were excluded from the primary outcome analysis. For secondary outcomes, FBG data were available for 103 participants because 1 participant did not complete end point FBG assessment. BMI and waist circumference data were available for 103 participants because 1 participant did not submit end point anthropometric measurements. LDL-C and HDL-C data were available for 102 participants because values could not be calculated in 2 participants due to elevated triglyceride levels. Reasons for dropout included nonstudy-related injury, pregnancy, personal commitments, and one nondiabetes-related hospitalization.

Table 1 summarizes participant baseline characteristics. The mean age of participants was 43.0 (SD 9.3) years, and 45.1% (55/122) of participants were male. The distribution of participants by age group and sex is presented in Multimedia Appendix 1. Most participants had been diagnosed with T2DM for less than 10 years (92/122, 75.4%). Most participants were receiving oral hypoglycemic therapy in addition to lifestyle management.

**Table 1.** Baseline demographics of participants (N=122).

Characteristic	Value
Age (y), mean (SD)	43.0 (9.3)
Sex, n (%)	
Male	55 (45.1)
Female	67 (54.9)
Duration of T2DM^a^, n (%)	
Diagnosis of <10 y	92 (75.4)
Diagnosis of ≥10 y	30 (24.6)
Treatment regimen, n (%)	
Lifestyle management only	3 (2.5)
Lifestyle management and 1 OHA^b^	31 (25.4)
Lifestyle management and 2 OHA	47 (38.5)
Lifestyle management and ≥3 OHA	30 (24.6)
Missing medication data	11 (9)

a T2DM: type 2 diabetes mellitus.

b OHA: oral-hypoglycemic agent.

Sex-stratified baseline anthropometric characteristics are additionally summarized in Multimedia Appendix 2.

### Primary Outcome

Among 104 participants with complete HbA_1c_ data, mean HbA_1c_ decreased from 8.8% (SD 1.2) at baseline to 7.6% (SD 1.1) following the 16-week intervention. This represented a mean reduction of −1.2% (95% CI −1.43 to −0.93; *P*<.001). A total of 65.4% (68/104) of participants achieved the predefined HbA_1c_ reduction threshold of ≥0.6% (95% CI 55.4%‐74.4%). Table 2 summarizes the primary HbA_1c_ outcomes following the intervention.

**Table 2.** Primary glycemic outcomes following the intervention (N=104).

Category	Value
Participants achieving HbA_1c_^a^ reduction ≥0.6%, n (%)	68 (65.4)
Participants with overall HbA_1c_ reduction, n (%)	88 (84.6)
Mean change in HbA_1c_, % (95% CI; *P* value)	−1.2 (95% CI −1.43 to −0.93; *P*<.001)

a HbA_1c_: glycated hemoglobin.

Overall, 65.4% (68/104) of participants achieved the predefined HbA_1c_ reduction threshold of ≥0.6%. Additionally, 84.6% (n=88) of participants demonstrated an overall reduction in HbA_1c_ following the intervention. A total of 1.9% (n=2) of participants had no change, and 13.5% (n=14) of participants had an increase in HbA_1c_ following the intervention.

**Figure 2.**
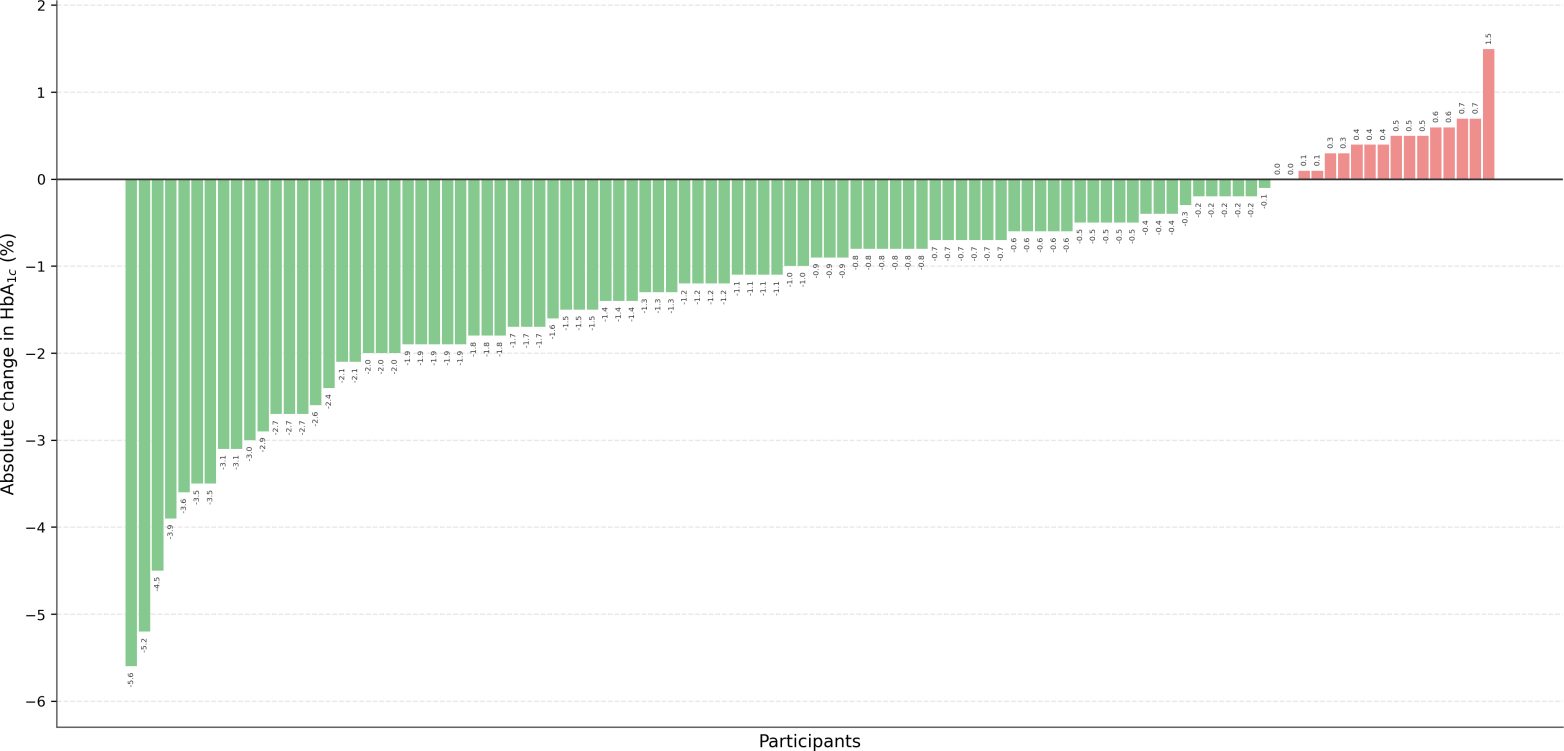
Individual changes in glycated hemoglobin (HbA_1c_) levels following the 16-week intervention (N=104). Negative values indicate a reduction in HbA_1c_ from baseline, while positive values indicate increases in HbA_1c_.

Individual changes in HbA_1c_ following the intervention are illustrated in Figure 2, demonstrating that most participants experienced reductions in HbA_1c_ levels after the 16-week intervention.

### Secondary Outcomes

#### Metabolic Outcomes

Significant improvements were observed in FBG, total cholesterol, and triglycerides. Table 3 summarizes changes in metabolic outcomes following the intervention.

**Table 3.** Changes in metabolic outcomes following the 16-week intervention.

Outcomes	n	Baseline, mean (SD)	End point, mean (SD)	Change, mean (SD)	95% CI for change	*P* value
FBG^a^, mmol/L	103^b^	9.3 (2.9)	7.6 (2.2)	−1.7 (2.8)	−2.3 to −1.2	<.001
Total cholesterol, mmol/L	104	4.9 (1.0)	4.6 (0.8)	−0.3 (1.0)	−0.6 to −0.2	<.001
HDL-C^c^, mmol/L	102^b^	1.2 (0.2)	1.1 (0.2)	−0.02 (0.14)	−0.1 to 0.00	.10
LDL-C^d^, mmol/L	102^b^	2.9 (0.8)	2.7 (0.7)	−0.2 (0.8)	−0.3 to 0.0	.14
Triglycerides, mmol/L	104	1.9 (1.7)	1.5 (0.8)	−0.5 (1.3)	−0.7 to −0.2	<.001

a FBG: fasting blood glucose.

b One participant did not complete the end point FBG assessment. LDL-C and HDL-C values were unavailable for 2 participants because values could not be calculated due to elevated triglyceride levels.

c HDL-C: high-density lipoprotein cholesterol.

d LDL-C: low-density lipoprotein cholesterol.

Among 103 participants with complete FBG data, mean FBG decreased significantly from 9.3 (SD 2.9) mmol/L at baseline to 7.6 (SD 2.2) mmol/L at week 16, representing a mean reduction of 1.7 mmol/L (95% CI −2.26 to −1.17; *P*<.001).

Lipid profile analyses demonstrated significant improvements in total cholesterol and triglyceride levels. Mean total cholesterol decreased from 4.9 (SD 1.0) mmol/L to 4.6 (SD 0.8) mmol/L, representing a mean reduction of 0.3 mmol/L (95% CI −0.55 to −0.17; *P*<.001). Mean triglyceride levels decreased from 1.9 (SD 1.7) mmol/L to 1.5 (SD 0.8) mmol/L, corresponding to a mean reduction of 0.5 mmol/L (95% CI −0.69 to −0.20; *P*<.001).

Reductions in LDL-C and HDL-C were also observed; however, these changes did not meet the predefined threshold for statistical significance.

#### Anthropometric Outcomes

Significant improvements in anthropometric measures were observed following the intervention (Table 4).

**Table 4.** Changes in anthropometric outcomes following the 16-week intervention.

Outcomes	n	Baseline, mean (SD)	End point, mean (SD)	Change, mean (SD)	95% CI for change	*P* value
BMI, kg/m^2^	103	33.0 (6.6)	32.6 (6.8)	−0.4 (1.0)	−0.6 to −0.2	<.001
Waist circumference, cm	103	105.4 (14.2)	103.5 (14.9)	−1.9 (5.2)	−2.9 to −0.9	<.001

Mean BMI decreased from 33.0 (SD 6.6) kg/m² to 32.6 (SD 6.8) kg/m², representing a mean reduction of 0.4 kg/m² (95% CI −0.60 to −0.22; *P*<.001). Mean waist circumference decreased from 105.38 (SD 14.17) cm to 103.49 (SD 14.91) cm, corresponding to a mean reduction of 1.89 cm (95% CI −2.90 to −0.89; *P*<.001). In sex-stratified analyses, male participants demonstrated a reduction in waist circumference from 108.7 (SD 14.4) cm to 106.8 (SD 14.1) cm, corresponding to a mean reduction of 1.9 cm (95% CI −2.93 to −0.94; *P*<.001). Female participants demonstrated a reduction from 104.7 (SD 14.1) cm to 102.8 (SD 15.3) cm, corresponding to a mean reduction of 1.9 cm (95% CI −3.59 to −0.13; *P*=.036), although this did not meet the predefined study significance threshold of *P*<.001.

Sex-stratified changes in BMI and waist circumference are summarized in Multimedia Appendix 3.

#### QoL Outcomes

Among 101 participants with complete QoL data, mean EQ-VAS scores improved significantly from 79.4 (SD 16.6) at baseline to 86.1 (SD 12.2) after the intervention. This represented a mean increase of 6.7 points (95% CI 3.9 to 9.6; *P*<.001).

Improvements were additionally observed across all EQ-5D-5L domains following the intervention, with higher proportions of participants reporting “no problems” at end point assessment compared with baseline assessment (Table 5). The largest improvements were observed in the pain/discomfort domain, from 65.3% (66/101) of participants at baseline to 82.1% (83/101) of participants at end point, and in the anxiety/depression domain, from 67.3% (68/101) of participants at baseline to 80.2% (81) of participants at end point. Under the predefined study statistical threshold of *P*<.001, none of the EQ-5D-5L domain improvements reached statistical significance, although the pain/discomfort domain showed the lowest *P* value (*P*=.002).

**Table 5.** EQ-5D-5L domain outcomes at baseline and end point.

EQ-5D-5L dimension	No problems at baseline, n (%)	No problems at end point, n (%)	*P* value
Mobility	90 (89.1)	98 (97)	.02
Self-care	99 (98)	100 (99)	>.99
Usual activities	87 (86.1)	95 (94.1)	.02
Pain/discomfort	66 (65.3)	83 (82.1)	.002
Anxiety/depression	68 (67.3)	81 (80.2)	.04

#### Engagement and Feasibility Outcomes

Participant engagement throughout the intervention remained high. Of the 122 participants enrolled, 108 (88.5%) participants completed the 16-week intervention, while 10 (8.2%) participants withdrew from the study and 4 (3.3%) participants did not attend any health coach VCs.

Engagement was additionally supported through structured reminder workflows, including weekly WhatsApp reminders for diary card submission, consultation reminders prior to scheduled appointments, and ongoing asynchronous communication with health coaches throughout the intervention period.

Among participants who completed the intervention, 64.8% (70/108) of participants attended all 7 scheduled VCs, while 75.9% (82/108) of participants attended at least 5 VCs throughout the intervention period. No intervention-related serious adverse events were reported throughout the study (Table 6).

**Table 6.** Engagement and feasibility outcomes (N=122).^a^

Category	Result, n (%)
Participants completing intervention	108 (88.5)
Withdrawals	10 (8.2)
Did not attend any VC^b^	4 (3.3)
Attended all 7 VCs	70 (64.8)
Attended ≥5 VCs	82 (75.9)

a Percentages for participants completing intervention, withdrawals, and participants who did not attend any video consultations were calculated using the total enrolled population as denominator (N=122). Percentages for attendance at video consultations were calculated using participants who completed the intervention as the denominator (N=108).

b VC: video consultation.

### Participant Feedback and Intervention Acceptability

Postintervention feedback from 91.7% (99/108) of participants who completed the intervention was analyzed (Table 7). The feedback questionnaire assessed study satisfaction, intervention duration, achievement of SMART goals, usefulness of learning materials, diabetes self-management, and experiences with health coaches. Participant feedback on study satisfaction and intervention duration was assessed using a 5-point Likert scale (1=extremely dissatisfied, 2=dissatisfied, 3=neutral satisfaction, 4=satisfied, and 5=extremely satisfied).

**Table 7.** Answers reported by participants in the participant feedback and intervention acceptability survey (N=99).

Category	Participants, n (%)
Satisfied/extremely satisfied with intervention	86 (86.9)
Intervention helped achieve SMART^a^ goals	83 (83.8)
Dietary recommendations useful	84 (84.8)
Online consultations useful	82 (82.8)
Improved understanding of diabetes self-management	86 (86.9)
Improved eating habits	85 (85.9)
Increased confidence in blood glucose self-monitoring	77 (77.8)
Adoption of a more physically active lifestyle	69 (69.7)
Health coach advice very helpful	92 (92.9)

a SMART: Specific, Measurable, Achievable, Relevant, and Time-bound.

Overall satisfaction with the intervention was high, with 86.9% (86/99) of participants reporting that they were satisfied or extremely satisfied with the study. Most participants (n=83, 83.8%) reported that the intervention helped them achieve SMART goals related to diabetes self-management. Dietary recommendations (n=84, 84.8%) and online consultations with health coaches (n=82, 82.8%) were identified as the most useful intervention components.

Participants additionally reported improvements in multiple self-management behaviors, including improved understanding of diabetes self-management (n=86, 86.9%), improved eating habits (n=85, 85.9%), increased confidence in self-monitoring blood glucose levels (n=77, 77.8%), and adoption of a more physically active lifestyle (n=69, 69.7%).

Most participants (n=92, 92.9%) also reported that advice provided by health coaches was very helpful in supporting diabetes self-management.

## Discussion

### Principal Findings

This pilot trial provides preliminary evidence supporting the feasibility and potential effectiveness of a hybrid digital health intervention for individuals with T2DM in Brunei Darussalam. Participation in the 16-week intervention was associated with significant improvements in glycemic control, anthropometric measures, lipid parameters, and health-related QoL. Participants additionally demonstrated high engagement, intervention completion, and overall satisfaction with the study.

The intervention was associated with a clinically meaningful reduction in HbA_1c_, with a mean reduction of 1.2 percentage points. Significant improvements were also observed in FBG, BMI, waist circumference, total cholesterol, triglyceride levels, and EQ-VAS scores. These findings suggest that comprehensive digital interventions integrating structured education, personalized coaching, and self-monitoring support may improve multiple aspects of diabetes management.

### Comparison With Prior Work

The improvements in glycemic control observed in this study compare favorably with existing literature evaluating digital interventions for T2DM management. A systematic review by Stevens et al [10] reported reductions in HbA_1c_ in 19 out of 20 intervention groups, with an average decrease of 0.9%. Similarly, Eberle et al [11] reported that disease-specific mHealth interventions were associated with improvements in glycemic outcomes, with an average HbA_1c_ reduction of 1.1% among individuals with T2DM.

A more recent systematic review and meta-analysis by Kerr et al [16] additionally demonstrated that digital interventions incorporating personalized coaching, structured self-monitoring, and higher-intensity engagement strategies were associated with greater glycemic improvements and improved self-management behaviors. The present findings are consistent with this literature, particularly given the intervention’s incorporation of regular health coach interaction, asynchronous communication, structured educational support, and ongoing self-monitoring activities.

Several components of the intervention may have contributed to the observed outcomes. The study incorporated personalized coaching, SMART goal setting, weekly education reinforcement, and flexible communication between participants and health coaches. Previous evidence suggests that asynchronous communication may facilitate sustained patient engagement by allowing flexible interactions without the scheduling limitations of conventional in-person consultations [16]. Personalized coaching and goal setting may additionally support behavior change by promoting self-efficacy, accountability, and adherence to dietary and physical activity recommendations.

The intervention was additionally associated with significant improvements in FBG, BMI, waist circumference, total cholesterol, and triglyceride levels. The reductions observed in triglycerides may reflect the strong dietary and lifestyle modification components incorporated within the intervention, including structured nutritional guidance, dietary self-monitoring, and continuous behavioral reinforcement from health coaches. Previous reviews evaluating digital lifestyle interventions have similarly reported improvements in metabolic and anthropometric outcomes following interventions incorporating dietary counseling and self-management support [17,18].

Although trends toward improvement were observed in HDL-C and LDL-C levels, these changes did not reach statistical significance. This may particularly reflect the pragmatic nature of the intervention, which emphasized general lifestyle modification and diabetes self-management support rather than rigidly structured exercise prescription. Physical activity recommendations were incorporated as part of general lifestyle counseling and health coaching, but no formal exercise prescription or supervised exercise program was implemented. Consequently, the intervention may not have provided sufficient exercise stimulus to elicit substantial changes in HDL-C and LDL-C levels. Future studies incorporating longer intervention periods and more intensive, structured exercise components may be needed to demonstrate significant improvements in these lipid outcomes.

The intervention was also associated with improvements in health-related QoL outcomes. Individuals living with T2DM frequently experience reduced QoL, impaired psychological well-being, and poorer social function, which may negatively affect long-term self-management and adherence to treatment recommendations [19]. Previous studies have demonstrated that behavioral and lifestyle interventions may improve both diabetes-related outcomes and QoL measures among individuals with T2DM [20,21].

In this study, participants demonstrated improvements across all EQ-5D-5L domains, with the largest improvements observed in the pain or discomfort and anxiety or depression domains. The significant improvement in EQ-VAS scores further supports participants’ perceived improvements in overall health status following the intervention.

### Feasibility and Acceptability

The findings additionally support the feasibility and acceptability of the intervention within a real-world national digital health setting. The intervention achieved a high completion rate of 88.5% (108/122), with most participants attending at least 5 scheduled VCs throughout the intervention period. High levels of participant satisfaction, perceived usefulness of dietary guidance, and positive experiences with health coaches were observed.

Participant engagement was supported through asynchronous communication workflows, weekly reminder systems, consultation reminders, and continuous access to health coaches throughout the intervention. These findings are important because many digital health studies primarily focus on glycemic outcomes while providing limited evaluation of participant engagement, acceptability, and perceived usefulness.

The high retention and engagement observed in this study suggest that integrating personalized coaching, structured reminders, and flexible digital communication platforms may support sustained participation in diabetes self-management interventions within routine care settings. However, despite generally positive engagement outcomes, adoption of more physically active lifestyles was comparatively lower than improvements in dietary behaviors, and self-management behaviors and understanding. This may reflect the challenges associated with sustaining exercise behavior change in real-world digital interventions. While participants received general physical activity guidance and encouragement throughout the intervention, the study did not incorporate structured exercise prescription, supervised exercise sessions, or individualized exercise training regimens. Future interventions may benefit from incorporating more structured exercise components to support long-term physical activity adherence and optimize cardiometabolic outcomes.

A major strength of this intervention was its integration within Brunei Darussalam’s existing national digital health ecosystem through the BruHealth platform. This approach enabled structured remote support while reducing reliance on frequent in-person consultations. Such hybrid digital care models may be particularly valuable in settings with constrained health care resources and increasing chronic disease burden.

### Strengths and Limitations

This study has several strengths. The intervention combined digital self-management support with personalized coaching and offline clinical support, reflecting a pragmatic real-world implementation model. The study additionally evaluated not only clinical outcomes but also QoL, engagement, feasibility, and participant acceptability outcomes, providing a broader assessment of intervention effectiveness.

Several limitations should also be considered. First, the single-arm, nonrandomized design limits causal inference and precludes direct comparison with standard care. Second, the relatively short intervention duration limits evaluation of the long-term sustainability of observed improvements. Third, self-reported dietary intake, physical activity, and weight records may be subject to recall and reporting bias. Finally, exclusion of individuals receiving insulin therapy or with advanced diabetes-related complications may limit generalizability to the broader T2DM population.

### Implications for Practice and Future Research

The findings from this pilot study support the potential role of hybrid digital interventions in improving diabetes outcomes within national health systems. Integration of digital coaching and remote self-management support within existing health infrastructure may help optimize resource utilization while expanding access to structured diabetes care.

Future studies should include randomized controlled designs with longer follow-up periods to evaluate long-term sustainability, cost-effectiveness, and scalability of digital diabetes interventions. Further evaluation of patient engagement patterns and intervention adherence may additionally help identify the components most strongly associated with clinical improvement.

### Conclusions

This pilot study provides preliminary evidence that a hybrid digital health intervention integrating personalized coaching, structured education, and remote self-management may improve glycemic control, metabolic outcomes, health-related QoL, and patient engagement among individuals with T2DM in Brunei Darussalam.

Integration within the BruHealth platform demonstrates the potential scalability of digital diabetes management strategies within routine care settings. Further large-scale controlled studies with longer follow-up durations are warranted to evaluate long-term sustainability, implementation outcomes, and cost-effectiveness.

## Supplementary material

10.2196/94551 Multimedia Appendix 1 Population pyramid demonstrating the age and sex distribution of enrolled participants (N=122).

10.2196/94551 Multimedia Appendix 2 Baseline age and sex distribution of enrolled participants (N=122).

10.2196/94551 Multimedia Appendix 3 Sex-stratified changes in BMI and waist circumference.

## Abbreviations

DEsireD: Development and Exploration of Effectiveness and Feasibility of Digital Intervention for Type 2 Diabetes Mellitus
DTx: digital therapeutics
EQ-VAS: EuroQol Visual Analog Scale
FBG: fasting blood glucose
HbA_1c_: glycated hemoglobin
HDL-C: high-density lipoprotein cholesterol
LDL-C: low-density lipoprotein cholesterol
mHealth: mobile health
QoL: quality of life
SMART: Specific, Measurable, Achievable, Relevant, and Time-bound
T2DM: type 2 diabetes mellitus
VC: video consultation
