# Supplementary figures and images for: Evaluating the Impact and Practicality of a National Digital Intervention for Type 2 Diabetes Mellitus: Single-Arm Nonrandomized Pilot Trial
Source: JMIR Form Res. 2026 Jul 29;10:e94551. doi: 10.2196/94551 (PMC13419280; doi:10.2196/94551)

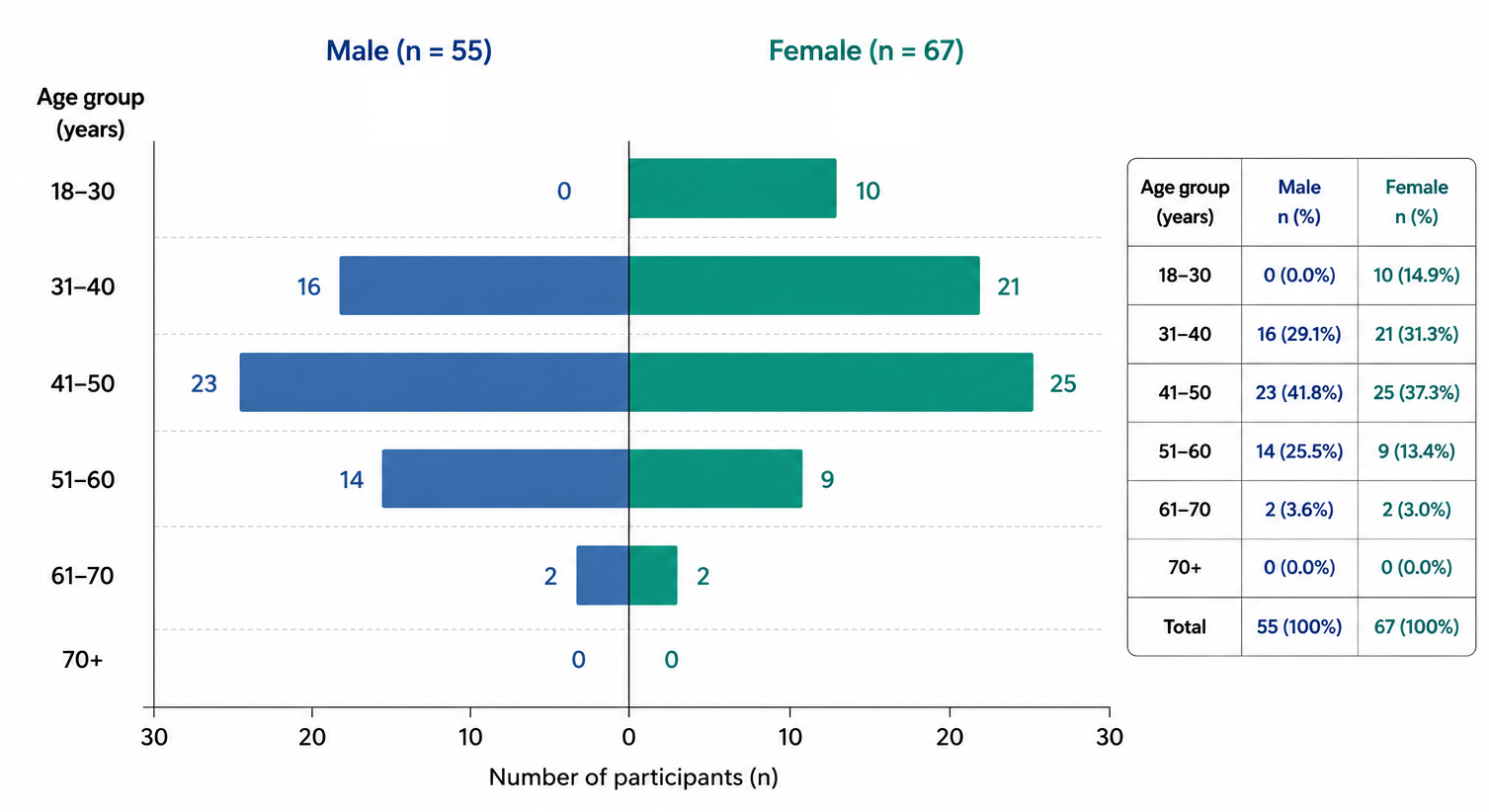

Supplement: Multimedia Appendix 1 [file formative-v10-e94551-s001.png]
