# Supplementary material for: Evaluating the Impact and Practicality of a National Digital Intervention for Type 2 Diabetes Mellitus: Single-Arm Nonrandomized Pilot Trial
Source: JMIR Form Res. 2026 Jul 29;10:e94551. doi: 10.2196/94551 (PMC13419280; doi:10.2196/94551)
Supplement: Multimedia Appendix 2 [file formative-v10-e94551-s002.pdf]

Multimedia Appendix 1. Baseline age and sex distribution of enrolled participants (n=122).

Characteristic	Male (n=55)	Female (n=67)
Age, years, mean (SD)	45.4 (8.1)	41.1 (9.8)
BMI, kg/m^2, mean (SD)	32.9 (6.3)	33.9 (6.8)
Waist circumference, cm, mean (SD)	108.7 (14.4)	104.7 (14.1)

BMI: body mass index.
