# Supplementary material for: Evaluating the Impact and Practicality of a National Digital Intervention for Type 2 Diabetes Mellitus: Single-Arm Nonrandomized Pilot Trial
Source: JMIR Form Res. 2026 Jul 29;10:e94551. doi: 10.2196/94551 (PMC13419280; doi:10.2196/94551)
Supplement: Multimedia Appendix 3 [file formative-v10-e94551-s003.pdf]

Multimedia Appendix 3. Sex-stratified changes in BMI and waist circumference

Outcome	Male (n=49)	Female (n=54)
BMI change, kg/m^2, mean (SD)	-0.4 (1.0)	-0.4 (1.0)
Weight change, kg, mean (SD)	-1.2 (2.8)	-0.9 (2.4)
Waist circumference change, cm, mean (SD)	-1.9 (3.5)	-1.9 (6.3)

BMI: body mass index.
